# Mode of delivery and child and adolescent psychological well-being: Evidence from Hong Kong’s “Children of 1997” birth cohort

**DOI:** 10.1038/s41598-017-15810-x

**Published:** 2017-11-15

**Authors:** Cherry Y. Leung, Gabriel M. Leung, C. Mary Schooling

**Affiliations:** 1 School of Public Health, Li Ka Shing Faculty of Medicine, The University of Hong Kong, Hong Kong, SAR People’s Republic of China; 2 Department of Community Health Systems, School of Nursing, UCSF, San Francisco, California, USA; 3 CUNY School of Public Health and Health Policy, New York, USA

## Abstract

Mode of delivery (vaginal or cesarean section) is thought to affect gut microbiota, which in turn may affect psychological well-being. As such, mode of delivery is potentially a modifiable factor for psychological well-being. Here we examined the association of mode of delivery with child and adolescent psychological well-being. We used multivariable linear regression in a population-representative Hong Kong Chinese birth cohort, “Children of 1997,” to examine the adjusted associations of mode of delivery with behavioral problems assessed from parent-reported Rutter score at ~7 (n = 6294) and ~11 years (n = 5598), self-esteem assessed from self-reported Culture-Free Self-Esteem Inventory score at ~11 years (n = 6937) and depressive symptoms assessed from self-reported Patient Health Questionnaire-9 score at ~13 years (n = 5797). Cesarean Section (CS) was associated with children born in private hospitals, boys, and firstborns, higher maternal body mass index, higher maternal age, preeclampsia, higher socioeconomic position (SEP) and maternal birth in Hong Kong. CS was unrelated to behavior, self-esteem and depressive symptoms adjusted for infant characteristics (sex, gestational age, birthweight, parity and breast feeding), maternal characteristics (mother’s age and place of birth) and SEP. In a developed non-Western setting, mode of delivery was not clearly associated with childhood or early adolescent psychological well-being.

## Introduction

Childhood psychological well-being is a growing concern in public health as mental health issues often emerge in adolescence and extend throughout adulthood with many other related adverse effects affecting health and social functioning. Cesarean section (CS) is a potentially modifiable factor that may be associated with the child’s psychological well-being. A growing body of evidence suggests that mode of delivery affects the infant’s gut microbiota. Infants born vaginally acquire gut microbiota from the maternal vaginal and fecal microbiota while infants born by CS acquire their gut microbiota from the environment^1^. Infants born vaginally initially have more microbes from the Firmicutes family and fewer from the Bacteroidetes family^2–4^. Although gut microbiota eventually normalize^5^, early life is a sensitive period when exposures may have lifelong consequences. Children born via CS have a higher risk of allergies^6^, asthma^7^ and diabetes^8^ later in life, with differences in gut microbiota during early life as a possible explanation. Gut microbiota are also associated with psychological well-being, through neural, endocrine and immune pathways, i.e., the gut-brain axis, which may affect brain function and behavior^9^. The idea that the gut could affect psychological well-being has been recognized since the middle of the nineteenth century and is generally accepted in the biological sciences^10^, and has been shown in several animal studies^11–13^.

The association of mode of delivery with cognitive development has previously been examined, with no association found for intelligence^14,15^ or cognitive delay^15^, and inconsistent associations for behavioral disorders^16–20^ and autistic spectrum disorder (ASD)^21,22^. Two recent Swedish cohort studies reported associations of CS with higher risk of ASD and psychosis, but the lack of associations in the sibling-matched analyses indicated that the observations were likely due to unmeasured genetic and environmental (i.e., differences in gut microbiota at birth) factors^21,23^. Another Swedish sibling-matched study found that birth by CS was associated with higher risk of attention-deficit/hyperactivity disorder (ADHD), but only elective CS had positive sibling associations, thus also implying that findings were confounded^18^. Curran *et al*. (2015) reported null associations of mode of delivery with ADHD and ASD in the Millennium Cohort in the United Kingdom^19^. However, the association of mode of delivery with other indicators of psychological well-being, as self-esteem during childhood and adolescent depressive symptoms during early adolescence has not, to our knowledge, previously been examined. CS rates tend to be higher than recommended by the World Health Organization (WHO) and thus represent a modifiable intervention target. To clarify the role of mode of delivery in psychological well-being, we examined prospectively in a large population-representative birth cohort, Hong Kong’s “Children of 1997,” the association of mode of delivery with child and adolescent psychological well-being, including measures of behavior, self-esteem, and depressive symptoms.

## Results

**Figure 1:**
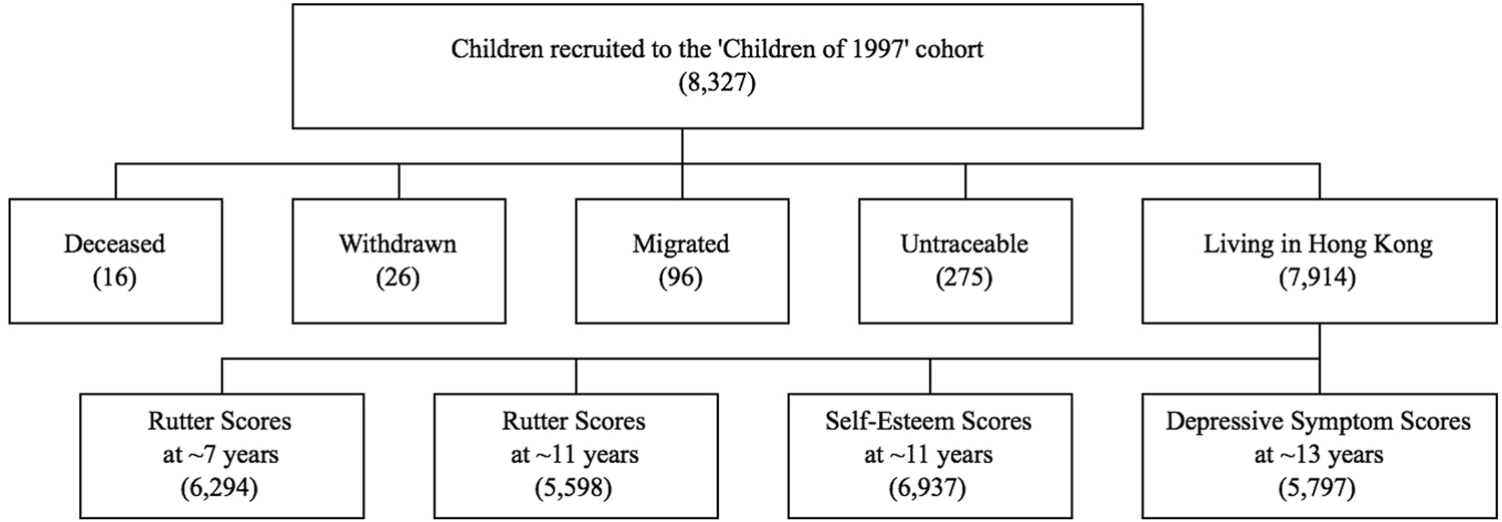
Hong Kong’s ‘Children of 1997’ birth cohort recruitment and follow-up (numbers) as of December 2013.

As shown in Fig. 1, of the original 8,327 cohort participants, as of December 2013, 16 (0.2%) were known to have died, 26 (0.3%) had permanently withdrawn, 96 (1.2%) were known to have migrated and 275 (3.3%) were untraceable, probably migrated, whereas 7,914 (95%) remained living in Hong Kong. Of these, 6,294 (80% follow-up) had parent-reported Rutter scores at ~7 years and 5,598 (71% follow-up) at ~11 years, 6,937 (88% follow-up) had self-reported self-esteem scores at ~11 years and 5,797 (73%) had depressive symptom scores at ~13 years.

About a quarter of the births were CSs (26.8%), 56.6% were unassisted vaginal birth and 16.6% assisted vaginal birth (Table 1). Private hospitals had higher rates of CS (48%) than public hospitals (18%). CS was positively associated with male sex, being firstborn, no breastfeeding, higher maternal BMI, higher maternal age, preeclampsia, higher SEP (parental education, occupation and household income at birth) and a Hong Kong-born mother.

**Table 1:** Baseline Characteristics by Mode of Delivery in Hong Kong’s “Children of 1997” Birth Cohort.

Characteristics	*N*	Model of Delivery	
		Vaginal % *(n* = 5,815)	Cesarean % *(n* = 2,143)
**Birth hospital**			
Public	5,430	79.6	48.4
Private	2,190	20.4	51.6
**Infant characteristics**			
Sex			
Girl	3,769	48.3	44.9
Boy	4,189	51.8	55.1
Birthweight (in grams)			
Mean (SD)	—	3,200 (400)	3,200 (500)
Gestational age (in weeks)			
Mean (SD)	—	39.1 (1.5)	38.9 (1.8)
Parity			
1	3,778	44.8	55.0
2	3,287	43.1	36.8
3 or above	878	12.1	8.3
Breastfeeding			
Never breastfed	4,518	54.4	63.3
Partially breastfed	2,954	38.6	33.3
Exclusively breastfed for ≥3 months	483	7.0	3.5
**Maternal characteristics**			
Mother’s birthplace			
Hong Kong	4,708	58.0	72.8
Non-Hong Kong	2,879	42.0	27.2
Maternal age at birth (in years)			
≤24	990	14.7	6.5
25–29	2,470	34.0	23.7
30–34	3,023	36.1	43.6
≥35	1,441	15.2	26.3
Maternal BMI			
Mean (SD)	—	22.4 (3.2)	23.1 (3.4)
Gestational diabetes			
Yes	382	6.5	7.1
No	5353	93.5	92.9
Preeclampsia			
Yes	279	4.4	6.2
No	5457	95.6	93.8
**Family SEP at birth**			
Highest parental education			
Grade 9 or below	2,392	32.7	23.2
Grade 10–11	3,388	42.7	42.5
Grade 12 or above	2,161	24.6	34.4
Highest parental occupation^a^			
I (professional)	1,684	22.8	29.4
II (managerial)	1,094	14.5	20.0
IIINM (non-manual skilled)	1,990	29.3	28.5
IIIM (manual skilled)	1,145	18.8	11.3
IV (semi-skilled)	696	11.2	7.6
V (unskilled)	226	3.4	3.2
Household income, dollars^b^ [mean (SD)]^a^			
1^st^ quintile [1,791 (432)]	1,358	21.9	12.9
2^nd^ quintile [2,913 (331)]	1,403	22.0	15.2
3^rd^ quintile [4,434 (538)]	1,397	20.0	20.2
4^th^ quintile 6,906 (887)]	1,394	18.4	24.4
5^th^ quintile [15,057 (15,852)]	1,418	17.8	27.3

Abbreviation: HK, Hong Kong; n, number; SD, standard deviation; SEP, socioeconomic position.

^a^At recruitment.

^b^In Hong Kong dollars (HK $7.80 = US $1.

Table 2 shows that mode of delivery was not associated with Rutter score or its subscores in any model. Mode of delivery was not associated with overall self-esteem, or most of its subscores, but was associated with lower social self-esteem. Mode of delivery was not associated with depressive symptoms in any model.

**Table 2:** Adjusted^a^ Associations of Mode of Delivery with Psychological Well-Being in Hong Kong’s “Children of 1997” Birth Cohort (Multiple Imputation).

Psychological Well-Being		Model 1^a^		Model 2^b^		Model 3^c^	
		Vaginal Delivery	Cesarean	Vaginal Delivery	Cesarean	Vaginal Delivery	Cesarean
		β	β (95% CI)	β	β (95% CI)	β	β (95% CI)
**Rutter scores at ~7 years**	(*n* = 6,294)						
Total		Ref	−0.30 (−0.63, 0.03)	Ref	−0.29 (−0.64, 0.05)	Ref	−0.27 (−0.63, 0.08)
Conduct		Ref	−0.03 (−0.10, 0.04)	Ref	−0.02 (−0.10, 0.05)	Ref	−0.02 (−0.10, 0.06)
Emotional		Ref	0.02 (−0.05, 0.08)	Ref	−0.04 (−0.11, 0.03)	Ref	−0.04 (−0.12, 0.03)
Hyperactivity		Ref	−0.11 (−0.20, −0.03)	Ref	−0.08 (−0.17, 0.01)	Ref	−0.08 (−0.17, 0.02)
**Rutter scores at ~11 years**	(*n* = 5,598)						
Total		Ref	−0.13 (−0.48, 0.22)	Ref	−0.03 (−0.40, 0.34)	Ref	−0.01 (−0.39, 0.38)
Conduct		Ref	−0.01 (−0.09, 0.07)	Ref	0.01 (−0.07, 0.09)	Ref	0.02 (−0.07, 0.10)
Emotional		Ref	0.05 (−0.03, 0.12)	Ref	0.0002 (−0.08, 0.08)	Ref	−0.01 (−0.09, 0.08)
Hyperactivity		Ref	−0.09 (−0.18, 0.001)	Ref	−0.02 (−0.11, 0.07)	Ref	−0.02 (−0.12, 0.08)
**Self-esteem scores**	(*n* = 6,937)						
Overall		Ref	0.54 (0.14, 0.95)	Ref	−0.24 (−0.65, 0.18)	Ref	−0.36 (−0.78, 0.07)
General		Ref	0.19 (0.003, 0.37)	Ref	−0.11 (−0.30, 0.09)	Ref	−0.15 (−0.35, 0.05)
Social		Ref	−0.22 (−0.30, −0.13)	Ref	−0.13 (−0.24, −0.02)	Ref	−0.12 (−0.23, −0.01)
Academic		Ref	0.20 (0.10, 0.31)	Ref	0.03 (−0.08, 0.14)	Ref	−0.02 (−0.13, 0.09)
Parent-related		Ref	0.14 (0.03, 0.25)	Ref	−0.03 (−0.14, 0.08)	Ref	−0.06 (−0.18, 0.05)
**Depressive symptoms**	(*n* = 5,797)						
PHQ-9 scores		Ref	−0.05 (−0.25, 0.14)	Ref	0.08 (−0.13, 0.29)	Ref	0.15 (−0.06, 0.37)

Abbreviation: HK, Hong Kong; CI, confidence interval; Ref, reference.

^a^Adjusted for sex, age at assessment and survey mode (PHQ-9 scores).

^b^Additionally adjusted for highest parental education, parental occupation, household income per head, mother’s birthplace and birth hospital.

^c^Additionally adjusted for birthweight, gestational age, parity, maternal age, maternal BMI, gestational diabetes and preeclampsia.

Table 3 shows that results were generally similar considering vaginal birth as unassisted and assisted. In a fully adjusted model (Model 3), CS compared to unassisted or assisted vaginal delivery was associated with lower social self-esteem. Results from a complete case analysis were similar (see Supplementary Tables S1 and S2).

**Table 3:** Adjusted^a^ Association of Mode of Delivery Separated by Unassisted and Assisted Vaginal Delivery with Psychological Well-Being in Hong Kong’s “Children of 1997” Birth Cohort (Multiple Imputation)

Psychological Well-Being	Model 1^a, b^		Model 2^a, c^		Model 3^a. d^	
	Assisted Vaginal Delivery	Cesarean	Assisted Vaginal Delivery	Cesarean	Assisted Vaginal Delivery	Cesarean
	β (95% CI)	β (95% CI)	β (95% CI)	β (95% CI)	β (95% CI)	β (95% CI)
**Rutter scores at ~7 years** (*n* = 6,294)						
Total	0.03 (−0.38, 0.43)	−0.29 (−0.63, 0.05)	0.08 (−0.34, 0.50)	−0.27 (−0.63, 0.10)	−0.12 (−0.55, 0.31)	−0.31 (−0.68, 0.07)
Conduct	−0.07 (−0.16, 0.02)	−0.04 (−0.12, 0.03)	−0.05 (−0.14, 0.04)	−0.04 (−0.12, 0.04)	−0.10 (−0.19, −0.003)	−0.05 (−0.13, 0.03)
Emotional	0.09 (0.01, 0.18)	0.04 (−0.03, 0.11)	0.05 (−0.04, 0.13)	−0.02 (−0.10, 0.05)	0.003 (−0.09, 0.09)	−0.04 (−0.12, 0.04)
Hyperactivity	−0.05 (−0.16, 0.05)	−0.13 (−0.22, −0.04)	0.001 (−0.11, 0.11)	−0.08 (−0.18, 0.01)	−0.03 (−0.14, 0.08)	−0.09 (−0.19, 0.01)
**Rutter scores at ~11 years** (*n* = 5,598)						
Total	−0.26 (−0.69, 0.17)	−0.20 (−0.57, 0.16)	−0.13 (−0.56, 0.31)	−0.07 (−0.47, 0.32)	−0.28 (−0.73, 0.17)	−0.11 (−0.52, 0.30)
Conduct	−0.03 (−0.12, 0.06)	−0.02 (−0.10, 0.06)	−0.002 (−0.10, 0.09)	0.004 (−0.08, 0.09)	−0.04 (−0.14, 0.06)	0.001 (−0.09, 0.09)
Emotional	0.03 (−0.06, 0.12)	0.06 (−0.02, 0.14)	−0.01 (−0.10, 0.09)	−0.002 (−0.09, 0.08)	−0.03 (−0.13, 0.07)	−0.01 (−0.10, 0.08)
Hyperactivity	−0.14 (−0.24, −0.03)	−0.12 (−0.22, −0.03)	−0.06 (−0.17, 0.05)	−0.04 (−0.14, 0.06)	−0.08 (−0.19, 0.04)	−0.05 (−0.15, 0.05)
**Self-esteem scores** (*n* = 6,937)						
Overall	1.42 (0.93, 1.90)	0.87 (0.45, 1.28)	0.51 (0.02, 1.00)	−0.10 (−0.53, 0.34)	0.29 (−0.21, 0.80)	−0.27 (−0.73, 0.18)
General	0.57 (0.35, 0.80)	0.32 (0.13, 0.51)	0.24 (0.01, 0.47)	−0.04 (−0.24, 0.16)	0.15 (−0.09, 0.38)	−0.11 (−0.32, 0.11)
Social	0.17 (0.05, 0.29)	0.05 (−0.05, 0.16)	−0.002 (−0.13, 0.12)	−0.13 (−0.24, −0.02)	−0.002 (−0.13, 0.13)	−0.12 (−0.24, −0.004)
Academic	0.39 (0.26, 0.52)	0.29 (0.18, 0.40)	0.19 (0.06, 0.32)	0.08 (−0.03, 0.20)	0.11 (−0.02, 0.25)	0.01 (−0.11, 0.13)
Parent-related	0.28 (0.15, 0.41)	0.20 (0.09, 0.31)	0.08 (−0.05, 0.21)	−0.01 (−0.13, 0.10)	0.04 (−0.10, 0.18)	−0.06 (−0.18, 0.06)
**Depressive symptoms** (*n* = 5,797)						
PHQ-9 scores	−0.26 (−0.50, −0.02)	−0.11 (−0.31, 0.09)	−0.15 (−0.40, 0.09)	0.04 (−0.17, 0.26)	−0.09 (−0.34, 0.16)	0.13 (−0.10, 0.35)

Abbreviation: HK, Hong Kong; CI, confidence interval

^a^Reference group: Unassisted vaginal delivery.

^b^Adjusted for sex, age at assessment and survey mode (PHQ-9 scores).

^c^Additionally adjusted for highest parental education, parental occupation, household income per head, mother’s birthplace and birth hospital.

^d^Additionally adjusted for birthweight, gestational age, parity, maternal age, maternal BMI, gestational diabetes and preeclampsia.

## Discussion

In this large, population-representative birth cohort from the developed non-Western setting of Hong Kong, consistent with one of two previous studies^16^, mode of delivery was not associated with child or adolescent behavior. Our study adds by showing CS was also not associated with depressive symptoms or overall self-esteem in adolescence, although CS was perhaps associated with lower social self-esteem.

“Children of 1997” is a large, population-representative birth cohort from a unique setting; nevertheless, our study has limitations. First, type of delivery was obtained by parent-report (mostly mothers), as opposed to clinical records, which could introduce measurement error. However, type of delivery was obtained shortly after delivery and is quite memorable. Second, screening tools were used to assess behavioral problems, self-esteem, and depressive symptoms as opposed to clinical diagnosis. However, these questionnaires have been validated in Chinese populations^24–26^, and our large sample size compensates for any lack of precision. Third, difficult and stressful births can lead to hypoxia and asphyxia resulting in neonatal encephalopathy and long-term neurodevelopmental impairments^27^. We do not know whether this occurred for any specific type of delivery. Fourth, we have no information regarding analgesic use during the prenatal and perinatal periods. Analgesic exposure during the fetal or neonatal period has been found to be associated with changes to the brain in animal studies^28,29^. However, no association has been found between obstetric anesthetic techniques and behavior or long-term neurodevelopmental outcomes in humans^30^. Fifth, we did not have data differentiating whether CS was elective or emergency. Infants born via emergency CS are typically exposed to the mother’s vaginal flora, as compared to elective CS, where infants are not^20^. A recent study reported that emergency CS, compared to spontaneous vaginal delivery, was associated with delayed gross motor function at 9 months and null associations across various modes of delivery with cognitive and motor development at 3 years^20^. However, there are no studies examining the longer term effects of emergency CS with psychological well-being. Finally, given the large number of associations examined, any positive associations might be false positives due to multiple comparisons and need to be confirmed in future studies.

Our study is not consistent with a link between gut microbiota, established early in life, and psychological well-being. In adults, probiotics, of the *Lactobacillus* or *Bifidobacterium* genera^31–33^, may have a role in the treatment of depression, as seen in some preliminary randomized controlled trials^31,32^. Differences may have occurred for a number of reasons. Mode of delivery may affect proportions of bacteria from the *Firmicutes* and *Bacteroidetes* families, but may only be associated with short-term differences in gut microbiota, rather than representing differences that continue into adolescence, although early life is a key phase for neurological development. Alternatively, any effects of events at birth may operate via different mechanisms, such as cortisol from a stress response^34–36^ leading to child behavioral problems. For example, Li *et al*. (2011) found elective cesarean delivery associated with fewer behavioral and emotional problems compared to unassisted vaginal delivery while assisted vaginal delivery was associated with more problems^17^. However, we found no associations of assisted vaginal delivery with behavioral problems. Furthermore, Li *et al*. (2011) did not examine the differences between the implications for CS, thereby not allowing further examination of how cortisol may mediate the pathway between mode of delivery and psychological well-being.

Mode of delivery does not appear to be associated with the psychological well-being of children and adolescents. However, a clearer understanding of the long-term effects of mode of delivery on gut microbiota will help elucidate the impact of CS on health, delineate any possible pathways involving the gut-brain-axis and identify where modification of gut microbiota in infants could potentially be beneficial.

## Methods

### Ethics Statement

Since our participants are children, informed consent was obtained from the parents, next of kin, caretakers or guardians (informants) on behalf of the participants by completing the questionnaire at enrollment as approved by The University of Hong Kong Medical Faculty Ethics Committee. Ethical approval for further studies was obtained from the University of Hong Kong-Hospital Authority Hong Kong West Cluster, Joint Institutional Review Board and/or the Ethics Committee of the Department of Health, Government of the Hong Kong SAR as appropriate. All methods were performed in accordance with the relevant guidelines and regulations.

### Participants

The Hong Kong “Children of 1997” birth cohort is a population representative Chinese birth cohort (n = 8,327) that covered 88.0% of all births in Hong Kong from April 1, 1997 to May 31, 1997, and has been described in detail elsewhere^37^. The study was initially established to investigate the effects of secondhand smoke exposure on infant health. Families were recruited at the first postnatal visit to one of the 49 Maternal and Child Health Centers (MCHCs) in Hong Kong, which parents of all newborns were encouraged to attend. Characteristics obtained using a self-administered questionnaire in Chinese at recruitment and subsequent routine visits included maternal characteristics (mother’s birthplace and age at birth), infant characteristics (type of delivery, type of birth hospital, sex, gestational age, birth weight, parity and breastfeeding) and socioeconomic position (SEP).

Passive follow-up via record linkage was instituted in 2005 to obtain routinely collected information including bi-annual assessments of emotional and behavioral problems using the Revised Parent’s Rutter Scales in Chinese (grade 2, 4 and 6 (i.e., ages 7-8, 9-10 and 11-12 years)) and self-esteem using Form A of the Culture-Free Self-Esteem Inventories for Children by Battle in Chinese (grade 4 (age 9-10 years) onwards) from the Student Health Service, Department of Health, which provides free annual check-ups for all school students. Active follow-up via direct contact was instituted in 2007, including postal surveys. Maternal height and weight, gestational diabetes and preeclampsia were obtained from surveys I (conducted in 2008 and 2009) and III (conducted in 2011 and 2012). Survey II, including the Patient Health Questionnaire-9 (PHQ-9), was first sent in February 2010 then re-sent a second and third time as necessary over the following 5 months. Non-responses were followed-up via two-waves of telephone interviews from November 2010 to April 2011 and from July 2011 to June 2012 and during pilot studies for in-person follow-up (June to August 2011 and June to August 2012). The study obtained ethical approval from the University of Hong Kong-Hospital Authority Hong Kong West Cluster Joint Institutional Review Board.

### Exposures

Mode of delivery was categorized as vaginal delivery or CS. Mode of delivery was obtained from the baseline survey completed by the primary caregiver (typically the mother). Mode of delivery was recorded as normal vaginal birth without instrumentation, vaginal birth with suction or forceps or CS. We considered vaginal births together (including unassisted and assisted vaginal delivery). Given that unassisted and assisted vaginal deliveries would be expected to have the same effect on gut microbiota, we also classified mode of delivery as unassisted vaginal delivery, assisted vaginal delivery or CS to check whether any associations were specific to CS. We did not collect information on the possible indications for CS or whether the CS was primary versus repeat.

### Outcomes

#### Emotional and behavioral problems

Emotional and behavioral problems at ~7 years (6- <9 years) and ~11 years (9- <13 years) were assessed from the Revised Parent’s Rutter Scales in Chinese for parents^25,38^. The scales consist of a set of 31 items describing emotional and behavioral difficulties, with each item scored 0 for *does not apply*, 1 for *applies somewhat* or 2 for *certainly applies*. A total score (range 0–62) and subscores for conduct problems (5 items, range 0–10), emotional problems (5 items, range 0–10) and inattention/hyperactivity (3 items, range 0–6) were calculated, where a higher score indicated more emotional and behavioral problems. When considering the presence of emotional and behavioral problems, a total score of 13 or more was considered as overall emotional and behavioral problems^39^. Presence of conduct problems, emotional problems or hyperactivity was defined as having the corresponding subscore above the sex-specific 97th percentile (≥5 for boys and ≥4 for girls for conduct, ≥4 for boys and ≥4 for girls for emotion, 6 for boys and ≥5 for girls for hyperactivity) for this population.

#### Self-esteem

Self-esteem at ~11 years (9- <13 years) was assessed from the Form A of the Culture-Free Self Esteem Inventories (SEI) in Chinese for children^40^. Assessment inventories developed in Western countries may be less valid in non-Western settings. However, the items of the SEI were chosen with concern for content that is least sensitive to change across cultures^41^. The SEI has good validation in assessing self-esteem in adolescents and has been used in Hong Kong Chinese^42^. Responses are of the forced-choice variety that the respondent checks either “yes” or “no” for each item. A total score (range 0–50) and subscores for general self-esteem (perception of self-worth in general) (20 item, range 0–20), social self-esteem (perception of quality of relationships with peer) (10 items, range 0–10), academic self-esteem (perception of ability to academic success) (10 items, range 0–10), and parent-related self-esteem (perception of status at home) (10 items, range 0–10) were calculated, where a lower score indicated lower self-esteem. Based on the findings from the same study, we excluded unreliable scores with a lie scale of 2 or less (n = 158), although a lie scale of 5 or more had been used to indicate a lack of defensiveness and a reliable self-esteem inventories^40^. Presence of low self-esteem was defined as having a total score of 19 or less^40^. Each subscale score can be classified into one of five categories (‘very low,’ ‘low,’ ‘intermediate,’ ‘high’ or ‘very high’). A ‘very low’ subscale score indicated low self-esteem in the respective subscale category (≤7 for general self-esteem and ≤2 for social, academic and parent-related self-esteem)^40^.

#### Depressive symptoms

Depressive symptoms at ~13 years (12- ≤15 years) were assessed from the PHQ-9^43^ in Chinese. The PHQ-9 scale has good sensitivity and specificity in detecting depression in youth^44^, is an effective screening tool for the risk of depression among adolescents^45^ and has been validated in Chinese adolescents^46^. The scales consist of a set of 9 items describing symptoms and functional impairment, with each item scored 0 for *not at all*, 1 for *several days*, 2 for *more than half the days* or 3 for *nearly everyday*. A total PHQ-9 score (range 0–27) was calculated, where a higher score indicated more depressive symptoms. Presence of PHQ-9 Major Depressive Disorder was defined as having a total score of 11 or more mapping on the DSM-IV-TR^44^.

### Statistical analysis

We used multivariable linear regression to estimate the adjusted associations of mode of delivery with Rutter, SEI and depressive symptom scores from which estimated beta-coefficients with 95% confidence intervals (CIs) are presented. We adjusted for potential confounders, which are presented in three models. Model 1 adjusted for sex, age at assessment and survey mode for depressive symptoms since for PHQ-9 was obtained by postal questionnaire, telephone interview and in-person interview. Model 2 additionally adjusted for SEP (highest parental education at birth, highest parental occupation at birth and monthly household income per head at birth), mother’s birthplace and birth hospital. Model 3 additionally adjusted for birth weight, gestational age, parity, maternal age, maternal body mass index (BMI), gestational diabetes and preeclampsia.

Among the 7,681 birth cohort members included, 3.6% were missing mode of delivery. Overall, 13.2% of data points were missing, mainly maternal BMI (58.8% missing) and gestational diabetes (23.6%) because these were obtained from Survey I or Survey III. We used multiple imputation to predict missing values of exposures and confounders incorporating data on the primary outcomes (Rutter, SEI and PHQ-9 scores), exposures (mode of delivery), confounders and other factors potentially associated with mode of delivery based on a flexible additive regression model with predictive mean matching^47^ as multiple imputation is the gold standard in this situation^48^. We used the ‘Hmisc’ package in R 3.0.1 (R Development Care Team, Vienna, Austria) for the multiple imputation. We summarized the results from 20 imputed datasets into single estimated beta-coefficients with CIs adjusted for missing data uncertainty. The results were based off 20 imputations due to fraction of missing information (γ) and the preventable power falloff of less than 1%. We also performed a complete case analysis for comparison using Stata version 10 (Stata Corp, College station, Texas).

### Data Availability

Data are available upon request from the “Children of 1997” data access committee: aprmay97@hku.hk.

## Electronic supplementary material

Supplementary Tables
