# Supplementary material for: Mode of delivery and child and adolescent psychological well-being: Evidence from Hong Kong’s “Children of 1997” birth cohort
Source: Sci Rep. 2017 Nov 15;7:15673. doi: 10.1038/s41598-017-15810-x (PMC5688155; doi:10.1038/s41598-017-15810-x)
Supplement: Supplementary file 1 — Supplementary Tables [file 41598_2017_15810_MOESM1_ESM.doc]

**Mode of delivery and child and adolescent psychological well-being:**

**Evidence from Hong Kong’s “Children of 1997” birth cohort**

Cherry Y Leung, Gabriel M Leung, C Mary Schooling

**Supplementary Table S1** Adjusted^a^ Associations of Mode of Delivery with Psychological Well-Being in Hong Kong’s “Children of 1997” Birth Cohort (Complete Case Analysis)

|  |  | **Model 1^a^** | |  | **Model 2^b^** | |  | **Model 3^c^** | |
| --- | --- | --- | --- | --- | --- | --- | --- | --- | --- |
|  |  | **Vaginal**  **Delivery** | **Cesarean** |  | **Vaginal**  **Delivery** | **Cesarean** |  | **Vaginal**  **Delivery** | **Cesarean** |
| **Psychological Well-Being** |  | β | β (95% CI) |  | β | β (95% CI) |  | β | β (95% CI) |
| **Rutter scores at ~7 years** | (*n*=6,294) |  |  |  |  |  |  |  |  |
| Total |  | Ref | -0.29 (-0.62, 0.04) |  | Ref | -0.23 (-0.62, 0.15) |  | Ref | -0.15 (-0.75, 0.45) |
| Conduct |  | Ref | -0.03 (-0.10, 0.05) |  | Ref | -0.01 (-0.09, 0.07) |  | Ref | -0.03 (-0.16, 0.09) |
| Emotional |  | Ref | 0.02 (-0.05, 0.08) |  | Ref | -0.03 (-0.11, 0.05) |  | Ref | -0.04 (-0.16, 0.09) |
| Hyperactivity |  | Ref | -0.11 (-0.20, -0.03) |  | Ref | -0.08 (-0.18, 0.02) |  | Ref | -0.06 (-0.22, 0.10) |
| **Rutter scores at ~11 years** | (*n*=5,598) |  |  |  |  |  |  |  |  |
| Total |  | Ref | -0.14 (-0.49, 0.21) |  | Ref | 0.12 (-0.29, 0.53) |  | Ref | -0.06 (-0.70, 0.59) |
| Conduct |  | Ref | -0.01 (-0.09, 0.07) |  | Ref | 0.04 (-0.05, 0.13) |  | Ref | 0.02 (-0.11, 0.16) |
| Emotional |  | Ref | 0.05 (-0.02, 0.13) |  | Ref | 0.02 (-0.07, 0.11) |  | Ref | -0.06 (-0.20, 0.08) |
| Hyperactivity |  | Ref | -0.09 (-0.18, -0.01) |  | Ref | -0.001 (-0.10, 0.10) |  | Ref | -0.08 (-0.24, 0.08) |
| **Self-esteem scores** | (*n*=6,937) |  |  |  |  |  |  |  |  |
| Overall |  | Ref | 0.54 (0.14, 0.94) |  | Ref | -0.15 (-0.61, 0.31) |  | Ref | -0.02 (-0.73, 0.69) |
| General |  | Ref | 0.19 (0.01, 0.37) |  | Ref | -0.04 (-0.25, 0.18) |  | Ref | -0.04 (-0.37, 0.30) |
| Social |  | Ref | 0.02 (-0.08, 0.12) |  | Ref | -0.12 (-0.24, -0.01) |  | Ref | 0.06 (-0.12, 0.24) |
| Academic |  | Ref | 0.20 (0.10, 0.31) |  | Ref | 0.03 (-0.09, 0.15) |  | Ref | -0.01 (-0.19, 0.18) |
| Parent-related |  | Ref | 0.13 (0.02, 0.24) |  | Ref | -0.02 (-0.14, 0.10) |  | Ref | -0.03 (-0.23, 0.16) |
| **Depressive symptoms** | (*n*=5,797) |  |  |  |  |  |  |  |  |
| PHQ-9 scores |  | Ref | -0.05 (-0.24, 0.14) |  | Ref | 0.18 (-0.05, 0.41) |  | Ref | 0.17 (-0.19, 0.53) |

Abbreviation: HK, Hong Kong; CI, confidence interval; Ref, reference.

^a^Adjusted for sex, age at assessment and survey mode (PHQ-9 scores).

^b^Additionally adjusted for highest parental education, parental occupation, household income per head, mother’s birthplace and birth hospital.

^c^Additionally adjusted for birthweight, gestational age, parity, maternal age, maternal BMI, gestational diabetes and preeclampsia.

**Supplementary Table S2** Adjusted^a^ Associations of Mode of Delivery Separated by Unassisted and Assisted Vaginal Delivery with Psychological well-being in Hong Kong’s “Children of 1997” Birth Cohort (Complete Case Analysis)

|  | **Model 1^a, b^** | | |  | **Model 2^a, c^** | |  | **Model 3^a. d^** | |
| --- | --- | --- | --- | --- | --- | --- | --- | --- | --- |
|  | **Assisted Vaginal Delivery** | | **Cesarean** |  | **Assisted Vaginal Delivery** | **Cesarean** |  | **Assisted Vaginal Delivery** | **Cesarean** |
| **Psychological Well-Being** | β (95% CI) | | β (95% CI) |  | β (95% CI) | β (95% CI) |  | β (95% CI) | β (95% CI) |
| **Rutter scores at ~7 years** (*n*=6,294) | |  |  |  |  |  |  |  |  |
| Total | 0.03 (-0.38, 0.43) | | -0.29 (-0.63, 0.06) |  | 0.01 (-0.46, 0.48) | -0.23 (-0.64, 0.18) |  | 0.23 (-0.49, 0.94) | -0.08 (-0.72, 0.56) |
| Conduct | -0.07 (-0.16, 0.02) | | -0.04 (-0.12, 0.03) |  | -0.07 (-0.17, 0.03) | -0.03 (-0.12, 0.06) |  | 0.08 (-0.15, 0.16) | -0.03 (-0.17, 0.10) |
| Emotional | 0.09 (0.01, 0.18) | | 0.04 (-0.03, 0.11) |  | 0.01 (-0.09, 0.10) | -0.03 (-0.11, 0.05) |  | -0.09 (-0.24, 0.05) | -0.07 (-0.20, 0.06) |
| Hyperactivity | -0.05 (-0.16, 0.05) | | -0.13 (-0.22, -0.04) |  | -0.02 (-0.14, 0.11) | -0.08 (-0.19, 0.03) |  | 0.12 (-0.07, 0.31) | -0.02 (-0.19, 0.15) |
| **Rutter scores at ~11 years** (*n*=5,598) | |  |  |  |  |  |  |  |  |
| Total | -0.26 (-0.68, 0.17) | | -0.20 (-0.57, 0.16) |  | -0.19 (-0.69, 0.03) | 0.06 (-0.38, 0.50) |  | -0.19 (-0.95, 0.58) | -0.12 (-0.81, 0.57) |
| Conduct | -0.03 (-0.12, 0.07) | | -0.02 (-0.10, 0.06) |  | 0.001 (-0.11, 0.11) | 0.04 (-0.05, 0.14) |  | 0.03 (-0.14, 0.19) | 0.03 (-0.12, 0.18) |
| Emotional | 0.03 (-0.06, 0.13) | | 0.06 (-0.02, 0.14) |  | -0.02 (-0.13, 0.09) | 0.02 (-0.08, 0.11) |  | -0.12 (-0.29, 0.04) | -0.10 (-0.25, 0.05) |
| Hyperactivity | -0.14 (-0.25, -0.04) | | -0.13 (-0.22, -0.04) |  | -0.06 (-0.18, 0.06) | -0.02 (-0.13, 0.09) |  | 0.04 (-0.14, 0.23) | -0.07 (-0.24, 0.10) |
| **Self-esteem scores** (*n*=6,937) | |  |  |  |  |  |  |  |  |
| Overall | 1.43 (0.95, 1.92) | | 0.87 (0.46, 1.28) |  | 0.45 (-0.10, 1.01) | -0.02 (-0.51, 0.47) |  | 0.13 (-0.73, 0.99) | 0.02 (-0.74, 0.78) |
| General | 0.58 (0.36, 0.81) | | 0.32 (0.13, 0.52) |  | 0.21 (-0.04, 0.47) | 0.03 (-0.20, 0.25) |  | 0.11 (-0.30, 0.51) | -0.002 (-0.36, 0.36) |
| Social | 0.17 (0.05, 0.30) | | 0.06 (-0.05, 0.16) |  | -0.05 (-0.19, 0.09) | -0.14 (-0.27, -0.01) |  | 0.01 (-0.21, 0.23) | 0.06 (-0.13, 0.26) |
| Academic | 0.40 (0.27, 0.52) | | 0.29 (0.18, 0.40) |  | 0.17 (0.03, 0.32) | 0.08 (-0.05, 0.21) |  | -0.01 (-0.23, 0.21) | -0.01 (-0.21, 0.19) |
| Parent-related | 0.28 (0.15, 0.41) | | 0.20 (0.08, 0.31) |  | 0.12 (-0.03, 0.27) | 0.02 (-0.11, 0.15) |  | 0.02 (-0.21, 0.25) | -0.03 (-0.23, 0.18) |
| **Depressive symptoms** (*n*=5,797) | |  |  |  |  |  |  |  |  |
| PHQ-9 scores | -0.27 (-0.50, -0.03) | | -0.11 (-0.31, 0.09) |  | -0.10 (-0.38, 0.17) | 0.15 (-0.09, 0.39) |  | -0.09 (-0.52, 0.34) | 0.14 (-0.25, 0.53) |

Abbreviation: HK, Hong Kong; CI, confidence interval

^a^Reference group: Unassisted vaginal delivery.

^b^Adjusted for sex, age at assessment and survey mode (PHQ-9 scores).

^c^Additionally adjusted for highest parental education, parental occupation, household income per head, mother’s birthplace and birth hospital.

^d^Additionally adjusted for birthweight, gestational age, parity, maternal age, maternal BMI, gestational diabetes and preeclampsia.
